# Analysis of Genomic and Transcriptomic Data Revealed Key Genes and Processes in the Development of Major Depressive Disorder

**DOI:** 10.3390/ijms26199557

**Published:** 2025-09-30

**Authors:** Sergey M. Ivanov, Vladislav S. Sukhachev, Olga A. Tarasova, Alexey A. Lagunin, Vladimir V. Poroikov

**Affiliations:** 1 Department of Bioinformatics, Institute of Biomedical Chemistry, Moscow 119121, Russia; withstanding@yandex.ru (V.S.S.); olga.a.tarasova@gmail.com (O.A.T.); alexey.lagunin@ibmc.msk.ru (A.A.L.); vladimir.poroikov@ibmc.msk.ru (V.V.P.); 2 Department of Bioinformatics, Pirogov Russian National Research Medical University, Moscow 117513, Russia

**Keywords:** major depressive disorder, depression, genomics, transcriptomics, transcriptome-wide association study, genome-wide association study, pathway analysis, molecular mechanisms, master regulators

## Abstract

Major depressive disorder (MDD) is one of the most common diseases, affecting millions of people worldwide. Existing antidepressants do not allow sustainable remission to be achieved in many cases, probably due to insufficient understanding of the etiopathogenesis of MDD. The aim of this study was to identify the key genes, pathways, and master regulators associated with MDD based on a combination of genomic and transcriptomic data analyses. We performed a transcriptome-wide association study (TWAS) to identify the increase and decrease in transcription of particular genes that can be associated with MDD risk, the results of which were used to perform a pathway enrichment analysis that elucidated the pathways and processes associated with MDD. Besides changes in the metabolism of neurotransmitters, the association of some other processes with MDD was revealed, including changes in phospholipid and glycan metabolism, chromatin remodeling, RNA processing and splicing, and cell–extracellular matrix interaction. The transcriptomic analysis performed for brain regions mostly confirmed genome-level findings. The gene expression changes in the brain related to MDD were mostly sex-specific, and the transcription of many genes was changed in the opposite direction in males and females. Finally, master regulators were found, which are the proteins responsible for the transcriptional regulation of the revealed genes and represent the most important proteins contributing to MDD development.

## 1. Introduction

Major depressive disorder (MDD) is a widespread mental pathology that affects nearly 185 million people worldwide [1]. It is the third leading cause of disability worldwide [2], associated with 700,000 suicides per year [3]. Despite the prevalence of depressive disorders, current therapeutic strategies such as the use of various antidepressants are not effective enough and do not allow complete remission to be achieved in more than half of cases [4]. This situation can be explained by the insufficient understanding of the etiology and pathogenesis of MDD. MDD can be considered a complex disease caused by both genetic and environmental factors such as post-trauma conditions, negative life events, childhood trauma, and post-COVID syndrome. Twin studies revealed that the heritability rate of depression is about 40% [5]. The molecular basis of MDD is still unclear, and various hypotheses have been proposed, including monoamine, neurotrophic, cytokine, stress-induced, mitochondrial dysfunction and oxidative stress, and metabolome/kynurenine depression models [1,5,6,7,8,9,10]. The first suggested biological mechanism of MDD is a deficiency in monoamine levels, such as serotonin, noradrenaline, and dopamine, in the brain. Existing antidepressants target the monoamine system by modulating receptor activity and the processes of neurotransmitter synthesis, degradation, and transport. The role of other neurotransmitters in the etiopathogenesis of MDD, such as gamma-aminobutyric acid (GABA) and glutamate, as well as opioid signaling, is widely discussed [7,10]. The neurotrophic hypothesis of depression focuses on the deficiency in neurotrophins, which are responsible for the formation, support, and plasticity of neuronal networks. Brain-derived neurotrophic factor (BDNF) and its receptors seem to be a promising pharmacological target for MDD treatment [7,10,11]. The cytokine theory of MDD highlights the importance of inflammation in the development of depression. Dysregulation of both innate and adaptive immunity is known to be associated with the development and severity of MDD. It includes the elevation of pro-inflammatory cytokines, increases in innate immune cell types, and changes in the differentiation of T cells and other immune cell types [6]. The role of both central and peripheral immunity in the development of MDD is currently being discussed [12]. In particular, the brain contains microglia, which carry out macrophage-like functions, exhibit a broad spectrum of activation states upon receiving various stimuli, and are activated in many neurodegenerative and neuropsychiatric diseases including, MDD, where they contribute to pathology by promoting neuroinflammation. In certain conditions, peripheral immune cells can cross the blood–brain barrier and enter the brain parenchyma, generally producing damage. Increased levels of pro-inflammatory cytokines and their influence on brain functions also contribute to the development of MDD. Prolonged inflammatory cytokine activity perturbs multiple neuronal functions, including impairment of neurotransmitter signaling and disruption of the synthesis, reuptake, and release of neurotransmitters. Thus, the sustained immune response observed in infection, malignancy, or autoimmune diseases can promote the development of MDD by long-term action on the neural system of various cytokines. The most important cytokines in the context of MDD include tumor necrosis factor alpha; interleukins 1 beta, 2, 4, 6, and 10 and the interleukin 1 receptor antagonist; transforming growth factor beta; C-reactive protein; and others [6,9,12]. The use of various anti-inflammatory agents appears to alleviate the symptoms of depression. Several anti-inflammatory compounds have been proposed as potential treatments for depression, including antibodies targeting cytokines or cytokine receptors, nonsteroidal anti-inflammatory drugs, and the microglia inhibitor minocycline [6,9,12]. Stress exposure, particularly early in life, is a well-known risk factor for MDD. Stress activates the hypothalamus–pituitary–adrenal (HPA) axis with the increase in cortisol levels. Chronic stress with HPA overactivity changes synaptic activity, increases neuroinflammation, and disrupts the level of neurotrophins. In turn, inflammatory cytokines can activate the HPA axis and sympathetic nervous system. Importantly, the changes in pro-inflammatory cytokines and depression appear to be bidirectional, as chronic stressors and current depressive symptoms, both associated with neurophysiological changes (e.g., glucocorticoid resistance), were found to increase stress reactivity, including cytokine changes in response to stressful challenges [13]. It has been shown that modulation of the HPA axis, including glucocorticoid antagonists, can improve the state of patients with MDD [7]. The mitochondria theory of depression focuses on the potential links between mitochondria defects and MDD. Changes in the mitochondria structure and functions lead to decreased ATP production and the increased generation of free radicals with the development of oxidative stress in nervous system cells. These processes are linked to the alteration in brain structure, monoamine levels, inflammation, and neural plasticity [7]. Mitochondria are currently proposed as a promising target for the therapy of MDD. It was shown that supplements of coenzymes/substrates for the oxidative phosphorylation chain can restore normal levels of mitochondrial function and improve mood [10]. The nutritional/microbiotic effects on the brain and development of MDD, including the role of kynurenine metabolism, are also widely described [7].

Despite the significant progress made in this field, the understanding of MDD mechanisms is far from complete. An important way to study disease etiopathogenesis is related to the analysis of OMICs data. The most used OMICs data types in MDD research are genomics and transcriptomics. Genome-wide association studies (GWASs) are the main source of associations between single-nucleotide polymorphisms (SNPs) and diseases. In recent years, several GWASs related to MDD were conducted, which included hundreds of thousands of individuals. The largest GWASs include studies by Howard [14], Hyde [15], Levey [16], Meng [17], and Wray [18]. In 2019, Howard and colleagues also conducted a meta-analysis using data from their previous study and studies by Hyde and Wray [19]. The analysis relied on genomic data from 807,553 individuals (246,363 cases and 561,190 controls) and revealed 102 independent SNPs, 269 genes, and 15 gene sets associated with depression, including both genes and pathways associated with synaptic structure and neurotransmission [19]. The most significant genes included sortilin-related VPS10 domain-containing receptor 3 (SORCS3), neuronal growth regulator 1 (NEGR1), transcription factor 4 (TCF4), and Ras-related protein Rab-27B (RAB27B). The transcriptome-wide association study (TWAS) is an approach that elucidates the relationships between SNPs, associated with a disease, and quantitative trait loci (QTL), including expression QTL (eQTL). TWASs allow the prediction of gene expression changes in particular tissues caused by SNPs and potentially associated with disease. Several recent TWASs were related to MDD [20,21,22,23,24,25,26,27,28]. They usually include TWAS methods followed by the application of co-localization and pathway enrichment analyses. For instance, Lorenza Dall’Aglio and colleagues performed TWAS based on 21 tissue datasets related to the brain, blood, thyroid, adrenal, and pituitary glands. To characterize the identified associations, they performed co-localization, conditional, and fine-mapping analyses. As a result, 94 genes, including NEGR1, CTC-467M3.3, TMEM106B, LRFN5, ESR2, and PROX2, were identified [20]. Xiaoyan Li and colleagues identified 53 genes, 7 of which (B3GALTL, FADS1, TCTEX1D1, XPNPEP3, ZMAT2, ZNF501, and ZNF502) were associated with depression in two independent brain eQTL datasets, suggesting that these genes may represent promising candidate risk genes for depression [23]. An important drawback of these studies is that they did not include a systematic comparison of TWAS-derived genes with the differentially expressed genes (DEGs) identified in transcriptomic studies. Several transcriptomic studies related to MDD have been performed in recent years [29,30,31,32,33,34,35,36,37,38,39]. However, in contrast to other psychiatric disorders such as bipolar disorder and schizophrenia, MDD is not associated with significant changes in gene expression in various brain regions. For example, in a study by Thomas Lanz and colleagues, only 90 DEGs were identified across the frontal cortex, hippocampus, and striatum, in contrast to 3158 genes for schizophrenia and 244 genes for bipolar disorder [32]. This situation hinders the comprehensive investigation of MDD-related mechanisms at the transcriptome level. Nevertheless, a few genes and pathways were identified in previous studies. For instance, S.P. Pantazatos and colleagues identified 35 DEGs, including humanin-like-8 (MTRNRL8), interleukin-8 (IL8), serpin family H member 1 (SERPINH1), and chemokine ligand 4 (CCL4). The Gene Ontology and pathway enrichment analyses suggested a lower expression of genes involved in oligodendrocyte differentiation, regulation of glutamatergic neurotransmission, and oxytocin receptor expression, providing evidence for altered DNA-dependent ATPase expression in the suicide MDD subgroup [35]. An important finding derived from transcriptomic studies is that the MDD-related transcriptional changes are sex-specific [31,40,41,42]. For example, Benoit Labonté and colleagues provided a comprehensive characterization of male and female transcriptional profiles associated with MDD across six brain regions. The authors showed a major rearrangement of transcriptional patterns in MDD, with limited overlap between males and females. In particular, they identified that the down-regulation of the female-specific gene Dusp6 in the mouse prefrontal cortex mimicked stress susceptibility in females, but not males, by increasing ERK signaling and pyramidal neuron excitability [31].

In the current study, we carried out both genomic and transcriptomic analyses to identify key genes, pathways, and biological processes associated with MDD. We performed TWAS to identify genes along with the direction of their transcriptional changes associated with MDD risk, the results of which were used to perform pathway enrichment analysis, elucidating the pathways and processes associated with MDD. The careful analysis of the TWAS-derived genes with relatively high *p*-values that were involved in the identified pathways revealed a wide range of genes and processes associated with MDD. The genes and pathways obtained by TWAS were compared to DEGs and their associated pathways obtained by transcriptomic analysis to identify similarities and differences in MDD mechanisms at the genomic and transcriptomic levels. Finally, master regulators (MRs) that are responsible for the transcriptional regulation of the revealed genes and represent the most important proteins contributing to MDD development were identified.

## 2. Results

### 2.1. Single-Nucleotide Polymorphisms, Genes, Pathways, and Tissues Associated with Major Depressive Disorder

We used summary statistics from a meta-analysis by Howard and colleagues [19] to identify SNPs, genes, and gene sets associated with MDD. We identified 4625 SNPs with *p*-values less than 5 × 10^−8^ (Table S1) and mapped them to chromosome positions and human genes using the FUMA platform (https://fuma.ctglab.nl/, accessed on 27 May 2024) [43] and Ensembl VEP tool (https://www.ensembl.org/info/docs/tools/vep/index.html, accessed on 23 March 2024) [44] (see Section 4). ANNOVAR software (https://annovar.openbioinformatics.org/en/latest/, accessed on 27 May 2024), as part of the FUMA platform, provided data on functional consequences of SNPs. About 83% of the 4625 SNPs were mapped on the intergenic regions and introns of genes. Only 54 SNPs were mapped to the exons of genes. Using the VEP tool, SIFT and PolyPhen algorithms predicted only four SNPs, which may cause functionally significant amino acid changes, leading to changes in protein function. These SNPs include rs1042602 associated with an S/Y change at the 192 position of tyrosinase (TYR), rs13195509 associated with a V/M change at the 207 position of butyrophilin subfamily 2 member A1 (BTN2A1), and rs34788973 and rs61742093 associated with I/T and A/S changes at the 39 and 300 positions of olfactory receptor 2B2 (OR2B2), respectively.

**Figure 1:**
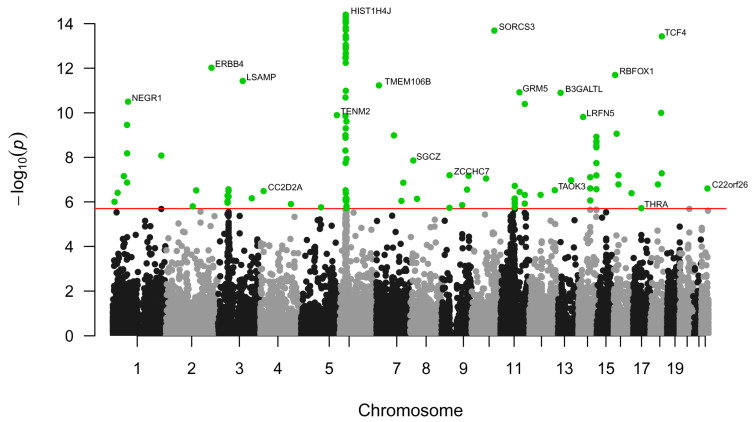
The Manhattan plot for genes identified by MAGMA. The x-axis represents the gene position on the chromosome; the y-axis represents the negative decimal logarithm of the *p*-value calculated by MAGMA. Green points refer to genes with *p*-values less than 0.05/19,081 = 2.62 × 10^−6^.

To identify genes associated with MDD using information on all SNPs regardless of their functional consequences, we used MAGMA software (version 1.08) as part of the FUMA platform [45]. MAGMA integrated *p*-values of all SNPs that are linked to the gene body and 10 megabases up- and downstream of the gene to obtain the gene-level *p*-value. According to the *p*-value threshold after Bonferroni correction, *p* = 0.05/19,081 = 2.62 × 10^−6^, we identified 112 genes related to MDD (Table S2). The most significant associations with MDD were found for genes from one region at the sixth chromosome (Figure 1). These genes included histones H1-5, H2AC13, H2BC13, H2BC15, H3C12, and H4C11 (all *p* < 1 × 10^−13^); butyrophilins BTN1A1, BTN2A1, BTN2A2, and BTN3A2 (all *p* < 1 × 10^−9^); transcription factors ZKSCAN4, ZKSCAN8, ZSCAN16, ZSCAN31, and ZSCAN9 (all *p* < 1 × 10^−11^); and some other genes: ABT1 (*p* < 2 × 10^−11^), NKAPL (*p* < 5 × 10^−14^), and PGBD1 (*p* < 4 × 10^−14^). Since these genes are in the same genomic risk loci (chromosome 6, positions 25684606-29448128), it is unclear which particular ones have causal relationships with MDD.

**Figure 2:**
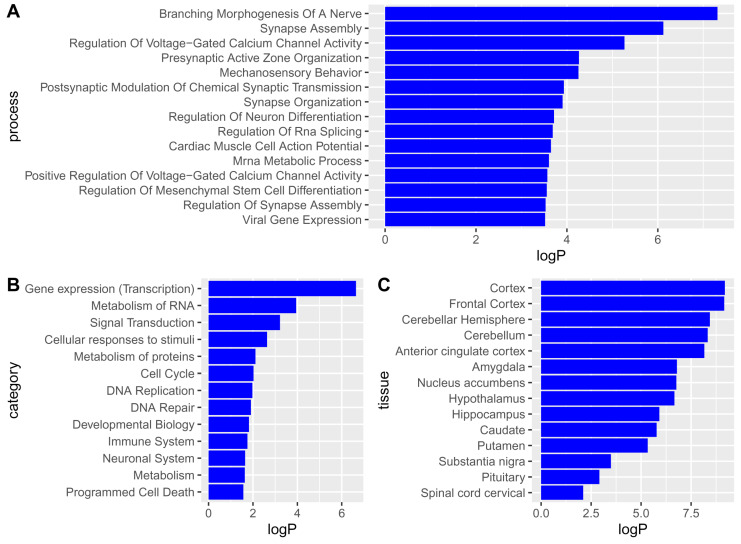
Results of gene-set enrichment analysis for genes identified by MAGMA: (**A**) top 15 Gene Ontology biological processes; (**B**) high-level Reactome pathways; (**C**) significant tissues from the GTEx database. logP is a negative decimal logarithm of the enrichment *p*-value.

Using the obtained gene-level statistics and MAGMA software, we identified Gene Ontology biological processes (GO BPs) (https://geneontology.org/, accessed on 27 May 2024), KEGG (https://www.genome.jp/kegg/pathway.html, accessed on 27 May 2024) and Reactome (https://reactome.org/, accessed on 27 May 2024) pathways, and GTEx human tissues (https://www.gtexportal.org/home/, accessed on 27 May 2024) related to MDD (Figure 2). The top GO BPs were mainly related to the functions and differentiation of neurons, including synaptic organization. By contrast, Reactome pathways were mainly related to gene expression processes, DNA replication and repair, cell survival and death, and processes in neural and immune systems. The role of these processes in MDD is discussed below. The complete list of the revealed pathways and biological processes is presented in Table S3.

Tissue enrichment analysis of the identified genes was also performed using MAGMA. The genes were associated with brain regions but not with non-brain tissues (Figure 2C). In particular, all brain regions available in GTEx were identified with *p*-values less than 0.05 (see Figure 2C), while other tissues had *p*-values greater than 0.05. Full data on the significance values obtained by MAGMA for human tissues is presented in Table S4.

Considering the tissue enrichment results, we performed TWAS using eQTL data for the brain regions only to obtain more details on genes and processes related to MDD (see Section 4).

### 2.2. Overview of Results from Transcriptome-Wide Association Study

We performed TWAS to identify gene transcription changes caused by SNPs in various brain regions potentially associated with MDD risk. We used S-PrediXcan software [46] (https://github.com/hakyimlab/MetaXcan, accessed on 24 April 2024) to calculate z-scores and *p*-values for thousands of genes for 15 brain regions. Positive (negative) z-scores obtained for a particular gene–brain region pair indicate that SNPs induce an increase (decrease) in transcription of the gene in the brain region, potentially increasing MDD risk. For further analysis, we selected gene–brain region pairs with *p*-values less than 0.05. It was found that the direction of transcription changes is the same across brain regions for almost all genes. Thus, we selected 684 genes that had either positive or negative z-scores and *p*-values < 0.05 in at least three brain regions. As a result, we predicted associations with MDD for the up-regulation of 346 genes and down-regulation of 338 genes (see Table S5). The increase (decrease) in transcription of these genes caused by SNPs may increase the predisposition and risk of MDD development.

Since the list of 684 genes is too large and may contain false positive associations with MDD, we performed a meta-analysis for 15 brain regions to calculate single *p*-values and account for the multiple testing problem (see Section 4). It was found that 371 out of 684 genes (54%) had an adjusted *p*-value (after Benjamini–Hochberg correction) less than 0.05. Among them, 187 genes were up-regulated, and 184 genes were down-regulated (see Table S5). Most of the 371 genes were identified in a wide range of brain regions and had the same direction of association with MDD, thus having a high probability of being associated with MDD.

In addition, we compared 371 genes with genes identified by colocalization analysis and Mendelian randomization. The corresponding data was imported from the COLOCdb database [47] (https://ngdc.cncb.ac.cn/colocdb/home, accessed on 18 September 2025). COLOCdb contains results of colocalization and Mendelian randomization analyses performed for a large number of GWASs, including the study by Howard and colleagues (study ID: P00965) used in our study. Colocalization analysis is considered to assess whether TWAS associations are driven by shared causal SNPs [48]. Twenty-six out of 371 genes were revealed by the colocalization analysis for at least one brain region, whereas an additional 9 genes (35 genes in total) were found for non-brain tissues (see Table S6). Mendelian randomization is a statistical method that uses SNPs as instrumental variables to infer the causal effect of a modifiable exposure (like eQTL) on a health outcome, such as MDD [49]. Sixty-six of the 371 genes were found in at least one brain region, while another 28 genes (94 genes in total) were found in non-brain tissues. HEIDI analysis showed that expression of 67 of 94 genes and MDD are potentially affected by the same underlying causal SNP (see Table S7).

We also compared the 371 genes obtained by TWAS in our study with genes identified in seven previous TWAS studies of MDD [16,17,18,20,22,23,26]. One hundred two of the 371 genes (27.5%) were identified in previous studies, while 269 genes were novel (72.5%) (see Table S8).

**Figure 3:**
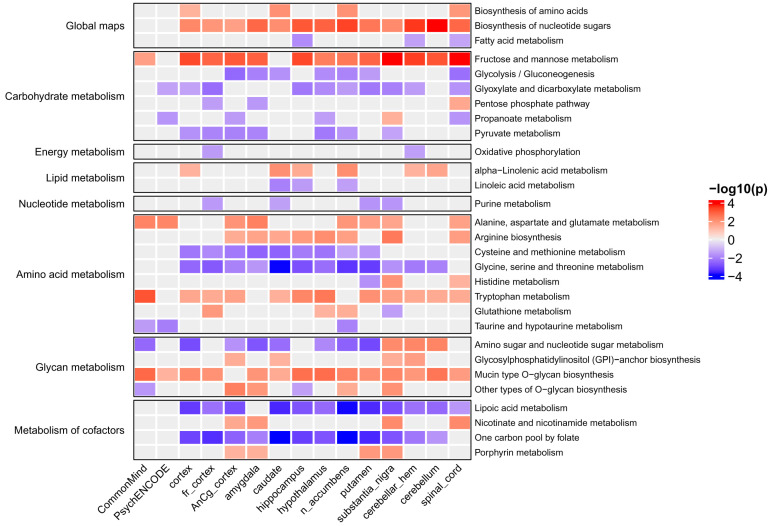
The revealed KEGG metabolic pathways based on TWAS-derived genes. The color of cells depends on the negative decimal logarithm of enrichment *p*-values. Red and blue colors refer to pathways enriched with up- and down-regulated genes, respectively.

**Figure 4:**
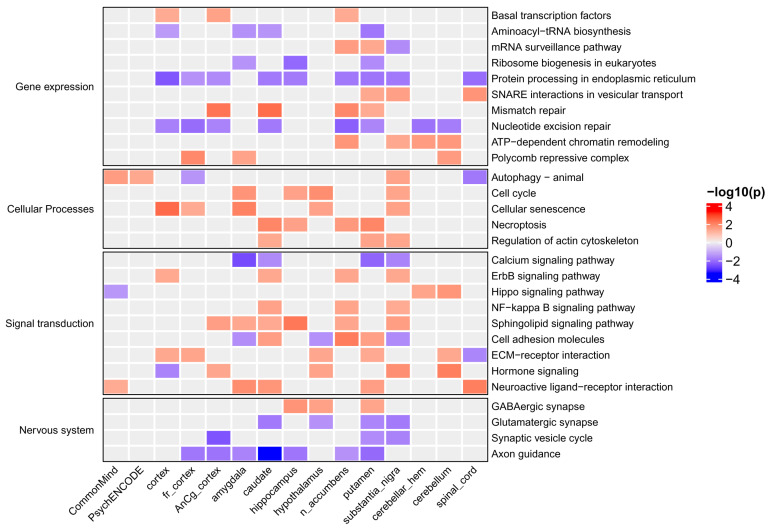
KEGG signaling pathways and cellular processes revealed based on TWAS-derived genes. The color of cells depends on the negative decimal logarithm of enrichment *p*-values. The red and blue colors refer to pathways enriched with up- and down-regulated genes, respectively.

To identify KEGG and Reactome pathways associated with MDD, we performed enrichment analysis (see Section 4) based on the obtained gene z-scores. Figure 3 and Figure 4 show KEGG signaling and metabolic pathways and cellular processes revealed in at least three brain regions. Importantly, in most brain regions, the activity of pathways changed in the same direction as for individual genes. The results of Reactome enrichment analysis are similar to those for KEGG and presented in Table S9. We manually estimated the positions of 371 genes in KEGG pathway maps as well as their known functions described in the UniProt database (https://www.uniprot.org/, accessed on 27 May 2024). This allowed us to group genes and pathways into several functional categories (see below). Table 1, Table 2 and Table 3 contain information on genes and their functions associated with MDD. Additionally, several genes identified in at least three brain regions with an unadjusted *p*-value < 0.05 (initial list of 684 genes, see above) were also included in the tables because they are parts of enriched pathways.

### 2.3. Metabolic Pathways Associated with Major Depressive Disorder

The many pathways identified based on the results of TWAS were related to cell metabolism (see Figure 3 and Table 1).

We suggested the following groups of metabolic pathways, enzymes, and metabolites whose modulation by SNPs can be associated with increased MDD risk.

**Energy metabolism**. Pathways “Glycolysis/Gluconeogenesis”, “Pyruvate metabolism”, and “Oxidative phosphorylation” were predicted to be down-regulated. Decreases in these processes lead to decreases in energy metabolism and decreases in the generation of ATP that may contribute to MDD [50].

**Nucleotide metabolism**. The down-regulation of pathways “Pentose phosphate pathway” and “Purine metabolism” is potentially associated with decreased nucleotide metabolism [51].

**Fatty acid metabolism**. Pathways “alpha-Linolenic acid metabolism”, “Linoleic acid metabolism”, and “Fatty acid metabolism” were linked to a decrease in fatty acid metabolism [51,52,53,54] and to changes in the metabolism of phospholipids [55,56].

**Amino acid metabolism**. The metabolism of some amino acids was related to the synthesis and degradation of neurotransmitters (see Table 1). The enzymes from KEGG pathways “Alanine, aspartate and glutamate metabolism”, “Arginine biosynthesis”, and “Glutathione metabolism” were associated with the increased synthesis of glutamate. The pathway “Taurine and hypotaurine metabolism” was down-regulated and associated with a decrease in taurine that, in turn, was associated with an increased risk of MDD [57,58,59]. “Glycine, serine and threonine metabolism” was linked to decreased degradation and an increased synthesis of glycine, which is a known co-agonist of glutamatergic receptors. Changes in tryptophan metabolism may be associated with changes in serotonin, kynurenine, and melatonin metabolism.

The pathways “Lipoic acid metabolism” and “One carbon pool by folate” contained enzymes that were also associated with glycine degradation. In addition, lipoic acid is a co-factor for many enzymes, essential for aerobic metabolism [60,61], which is also involved in glycine metabolism.

**Glycoprotein and glycolipid metabolism**. Glycan-metabolism-related pathways “Amino sugar and nucleotide sugar metabolism”, “Mucin type O-glycan biosynthesis”, “Other types of O-glycan biosynthesis”, “Fructose and mannose metabolism”, and “Glycosylphosphatidylinositol (GPI)-anchor biosynthesis” contain enzymes involved in protein glycosylation whose changes may be associated with MDD [62].

We list the corresponding enzymes from these pathways along with the TWAS-derived direction of their transcriptional changes and main functions in Table 1. Details regarding their role in MDD development are described in Section 3.

### 2.4. Signaling Pathways and Cellular Processes Associated with Major Depressive Disorder

We performed a similar analysis for signaling pathways from KEGG, as well as for pathways describing the regulation of gene expression and cellular processes. Identified pathways are shown in Figure 4, and the proteins (genes) from the pathways are shown in Table 2 and Table 3.

We found that MDD-related SNPs are potentially associated with the changes in all stages of gene expression: from transcription to protein transport. The corresponding processes are given below.

**DNA synthesis and repair**. We found that changes in DNA synthesis and repair can be associated with MDD. Most of the genes related to these processes (see Table 2) have negative TWAS z-scores, suggesting the potential link between the down-regulation of DNA repair enzymes and the increased risk of MDD [63,64].

**RNA processing and splicing**. The proteins associated with the splicing and processing of matrix, transport, and ribosomal RNA (mRNA, tRNA, and rRNA, respectively) were predicted to be associated with MDD [65].

**Chromatin regulation**. We identified various genes encoding proteins involved in chromatin remodeling and histone modification, e.g., components of the INO80 complex, Polycomb group proteins, histones, and their methyltransferases [66].

**Translation regulators and ribosome proteins**. The genes encoding ribosome proteins and the proteins required for translation were identified. Interestingly, the up-regulation of most of these genes was predicted to be associated with MDD [67,68,69].

**Vesicular transport**. The genes encoding proteins involved in vesicle-mediated transport, including transport from the endoplasmic reticulum to the Golgi, were predicted to be associated with MDD. Many of the revealed proteins are involved in neurotransmitter transport.

Besides processes related to gene expression, we identified several cellular processes associated with cell survival, death, cytoskeleton regulation, and cell adhesion to the extracellular matrix (ECM). We predicted SNP-induced changes in the transcription of genes from KEGG pathways describing cell growth, catabolism, and death: “Autophagy—animal”, “Cell cycle”, “Cellular senescence”, “Necroptosis”, and “Unfolded protein response”. Analysis of particular TWAS-derived genes revealed that cell cycle genes were predicted to be down-regulated, whereas genes associated with cell death induction were predicted to be up-regulated (see Table 2). For instance, the up-regulation of genes encoding unfolded protein response proteins, activating transcription factor 6 beta and glutamine-rich 1, as well as gasdermins D and E, may be associated with increased MDD risk [70,71].

The other group of pathways was related to cytoskeleton regulation and cell adhesion to the ECM. Cytoskeletal proteins encoded by TWAS-derived genes included several actin regulators and motor proteins such as myosins, kinesins, and dyneins. Besides the involvement of these proteins in other cellular processes such as mitosis, they participate in processes of anterograde and retrograde transport in neurons, including the transport of vesicles with neurotransmitters. Most of the kinesins, dyneins, and actin regulators were predicted by TWAS to be down-regulated [72,73]. Several genes encoding proteins participating in cell adhesion to the ECM were also identified. They include both membrane proteins and components of the ECM: integrins, protocadherins, collagens, and some other proteins (see Table 2) [74,75,76,77].

We also identified some signaling pathways, including the calcium, ErbB, Hippo, NF-kappa B, and sphingolipid signaling pathways. In our study, we focused on receptors and their ligands, since they are extremely important for communication between neurons and other cells. Table 3 shows the receptors, ligands, and ion channels that were revealed by TWAS analysis. Besides receptors for neurotransmitters, hormones, and growth factors, various immune proteins were identified. The possible role of the receptors and ligands in MDD is described in Section 3.

### 2.5. Differentially Expressed Genes and Associated Pathways in Post-Mortem Brain Tissues Revealed by Transcriptomic Analysis

We identified DEGs between samples from individuals with and without MDD for various brain regions (see Section 4). Taking into account the results from previous studies [31,40,41,42], we calculated DEGs for males and females separately (Table 4). As a result, using combinations of the GEO dataset and sex and brain region, we obtained 20 datasets for differential expression analysis. We considered a gene to be differentially expressed by applying soft and strict thresholds: a |logFC| more than 0.5 and an unadjusted *p*-value less than 0.05, and a |logFC| more than 1 and an adjusted *p*-value less than 0.05 (after Benjamini–Hochberg correction), respectively. We revealed few or no DEGs in most of the brain regions using a strict threshold, which suggests weak gene expression changes in MDD compared to healthy controls. Thus, in further analysis, we used the DEGs obtained by applying a soft threshold that allowed us to reveal dozens to hundreds of DEGs depending on the brain region (see Table 4).

**Figure 5:**
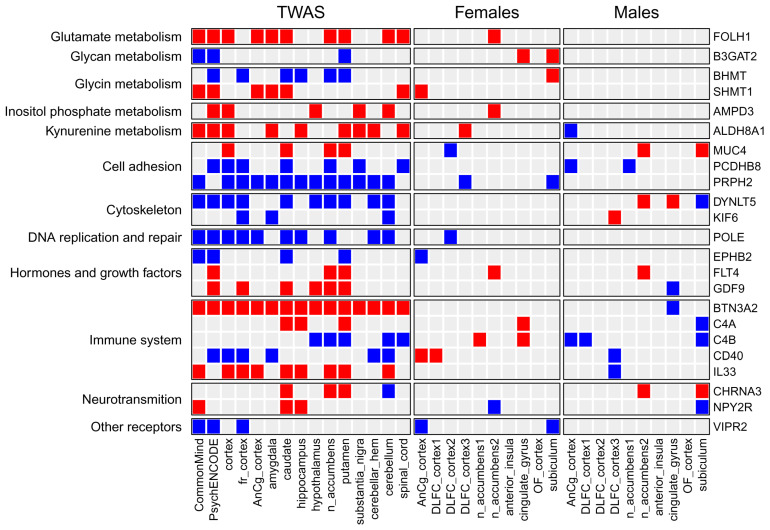
Comparison of 23 TWAS-derived genes with genes that were differentially expressed in various brain regions. The red and blue colors represent up- and down-regulated genes, respectively. DLFC_cortex1 and n_accumbens1 refer to the GSE80655 dataset, DLFC_cortex2 and n_accumbens2 refer to the GSE102556 dataset, and DLFC_cortex2 refers to the GSE101521 dataset.

We compared the list of TWAS-derived genes with the lists of DEGs. It was found that only 99, 40, 19, 9, 5, 4, and 2 out of 684 TWAS-derived genes were differentially expressed in at least 1, 2, 3, 4, 5, 6, and 7 of the 20 transcription datasets (see Table S10). Figure 5 demonstrates 23 TWAS-derived genes involved in MDD-related pathways (see Table 1, Table 2 and Table 3) that were differentially expressed in one or several brain regions from either males or females. The transcription of some of them, e.g., complements C4A and C4B and the CD40 receptor, was changed in different directions in males and females. The direction of SNP-induced gene expression changes predicted by TWAS was similar to gene transcription changes observed in either males or females depending on the gene. This can be explained by the fact that TWAS was performed based on eQTL data, which was obtained using transcription profiles from the joint male and female data.

**Figure 6:**
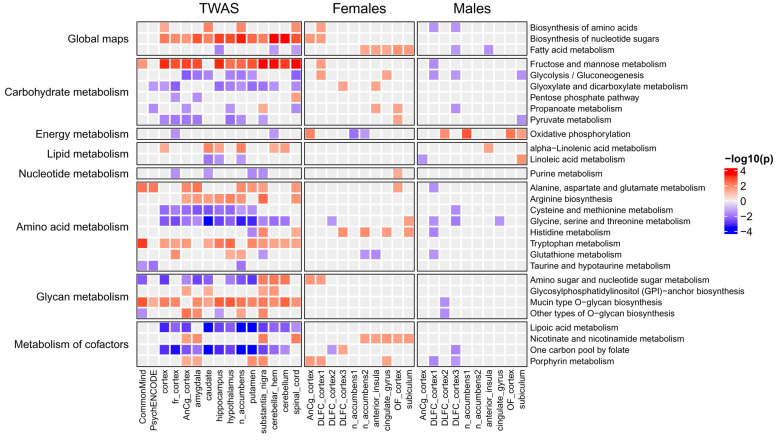
Comparison of KEGG metabolic pathways obtained by TWAS with pathways obtained by differential expression analysis. The color of cells depends on the negative decimal logarithm of enrichment *p*-values. The red and blue colors refer to pathways enriched with up- and down-regulated genes, respectively. DLFC_cortex1 and n_accumbens1 refer to the GSE80655 dataset, DLFC_cortex2 and n_accumbens2 refer to the GSE102556 dataset, and DLFC_cortex2 refers to the GSE101521 dataset.

**Figure 7:**
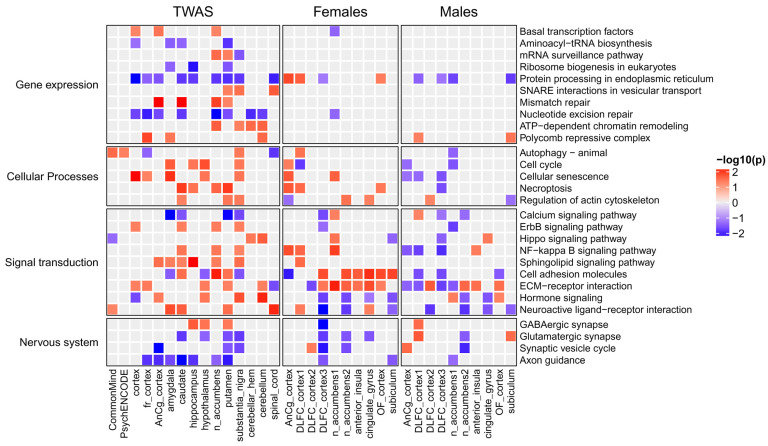
Comparison of KEGG signaling pathways and cellular processes obtained by TWAS with pathways and processes obtained by differential expression analysis. The color of cells depends on the negative decimal logarithm of enrichment *p*-values. The red and blue colors refer to pathways enriched with up- and down-regulated genes, respectively. DLFC_cortex1 and n_accumbens1 refer to the GSE80655 dataset, DLFC_cortex2 and n_accumbens2 refer to the GSE102556 dataset, and DLFC_cortex3 refers to the GSE101521 dataset.

**Figure 8:**
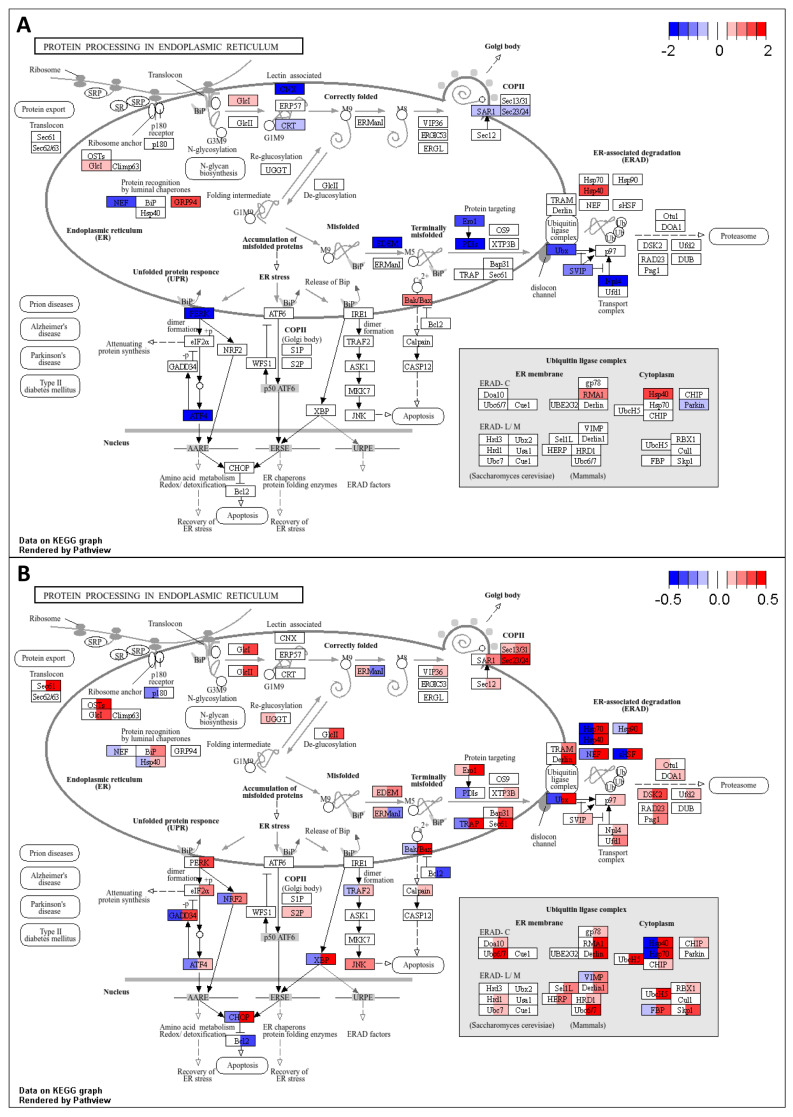
Comparison of the transcription changes in genes in the dorsolateral prefrontal cortex predicted by TWAS (**A**) and obtained by transcription analysis (**B**) that are involved in protein processing in the endoplasmic reticulum. The color of pathway nodes depends on either TWAS z-scores (**A**) or logFC values (**B**). The colors of the left and right parts of the pathway nodes depend on logFC values obtained for males and females, respectively (**B**). Generally, red and blue colors refer to up- and down-regulated genes, respectively. Part (**A**) of the figure was created using TWAS z-scores obtained using GTEx cortex data, while part (**B**) was created using logFC values obtained by analysis of dorsolateral prefrontal cortex data from the GSE80655 dataset.

The obtained information on gene transcription changes was used to identify KEGG pathways enriched by up- or down-regulated genes (see Section 4). The intersection between pathways enriched by TWAS-derived genes and pathways enriched by DEGs was larger than between the corresponding genes (see Figure 6 and Figure 7). We found that many metabolic pathways and cellular processes were identified only by TWAS or identified in a few brain regions by DEG analysis. The largest number of intersections was found for signaling pathways. The activity of many pathways changed in individuals of only one sex, whereas the activity of several pathways was changed in the opposite direction in males and females, e.g., glycolysis, oxidative phosphorylation, and protein processing in the endoplasmic reticulum. The “protein processing in endoplasmic reticulum” pathway is shown in Figure 8 as an example. The direction of transcription changes was opposite in males and females for many genes, as shown in previous studies [31,40,41,42]. The direction obtained by TWAS was generally similar to that obtained for males (see Figure 6 and Figure 7).

### 2.6. Identification of Master Regulators Responsible for the Observed Gene Expression Changes

**Figure 9:**
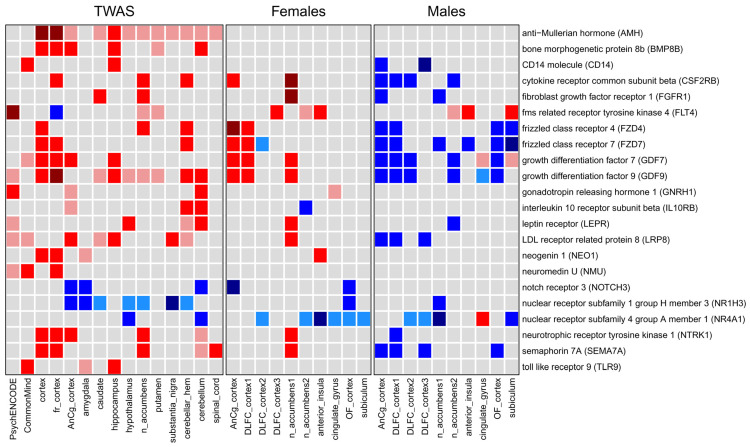
Master regulators that are potentially responsible for the transcription changes in TWAS-derived genes. If the master regulator was identified as “active” (“inactive”) in a particular brain region and the corresponding gene was also up-regulated (down-regulated) based on either TWAS or transcriptomics analysis, it was represented in a dark red (dark blue) color, while it was represented in a red (blue) color if the corresponding gene was not up-regulated (down-regulated). If the master regulator was not identified as “active” (“inactive”) in a particular brain region but the corresponding gene was up-regulated (down-regulated), it was represented in a light red (light blue) color.

We revealed that the transcriptional changes induced by SNPs potentially disrupted many cellular processes, leading to an increased MDD risk. We proposed that TWAS-derived genes may have common mechanisms of transcriptional regulation, including transcription factors and their upstream regulators, e.g., kinases, phosphatases, and receptors. These proteins are called master regulators (MRs), and they are usually responsible for the observed gene expression changes. We identified MRs based on the signaling network analysis approach (see Section 4). We identified “active” MRs, which activate the transcription of up-regulated genes and repress the transcription of down-regulated genes, and “inactive” MRs, which repress the transcription of up-regulated genes and activate the transcription of down-regulated genes. If “inactive” MRs were “active”, the transcription profile would change to a “normal”, healthy state. We performed this analysis for both transcription changes predicted by TWAS and logFC profiles obtained by transcriptomic analysis. We selected MRs that were differentially expressed based on at least one of two criteria: (1) the gene encoding the MR was revealed by TWAS in at least one brain region, and (2) the gene encoding the MR was differentially expressed in at least one post-mortem brain region. We identified 95 MRs following these criteria (see Table S11). Figure 9 shows information on the MRs, which are receptors and ligands, since they are crucially important for communication between neurons and between neurons and other cells in the brain.

We identified MRs mainly belonging to hormones, growth factors, and immune proteins. Some of the receptors and ligands are clearly linked to neural development, differentiation, and functions, such as growth differentiation factor 7 (GDF7), neogenin 1 (NEO1), neurotrophic receptor tyrosine kinase 1 (NTRK1), and semaphorin 7A (SEMA7A). Another group of MRs includes hormones involved in gonad differentiation, e.g., anti-Mullerian hormone (AMH), growth differentiation factor 9 (GDF9), and gonadotropin releasing hormone 1 (GNRH1). Immune MRs include the CD14 molecule (CD14), cytokine receptor common subunit beta (CSF2RB), interleukin 10 receptor subunit beta (IL10RB), and toll-like receptor 9 (TLR9). Note that many MRs were “active” in females but “inactive” in males, e.g., cytokine receptor common subunit beta (CSF2RB), frizzled class receptors 4 and 7 (FZD4 and FZD7, respectively), and growth differentiation factors 7 and 9 (GDF7 and GDF9, respectively) (see Figure 9). The potential role of these MRs in MDD is described in Section 3.

## 3. Discussion

We performed an analysis of genomic and transcriptomic data to identify key genes, pathways, and master regulators involved in MDD development. The analysis included several consequent steps: (1) identification of SNPs related to MDD and their mapping to human genes and pathways; (2) application of TWAS methods to estimate genes and pathways along with the direction of their expression changes that are associated with MDD risk; (3) identification of DEGs and their associated pathways in various brain regions based on RNA-seq data and their comparison with genes and pathways revealed by TWAS; (4) identification of MRs that are the proteins in the signaling network potentially responsible for the gene transcription changes observed in MDD.

### 3.1. Single-Nucleotide Polymorphisms Associated with Major Depressive Disorder and Their Mapping to Genes, Pathways, and Tissues

To identify the SNPs, genes, and gene sets associated with MDD, summary statistics from the meta-analysis by Howard and colleagues [19] were used. We revealed 4625 SNPs, most of which were mapped to the intergenic regions and introns of genes. MAGMA analysis revealed 112 genes significantly associated with SNPs related to MDD. We found that these genes participated in a wide range of processes, including gene expression, DNA replication and repair, cell survival and death, processes of neural differentiation and development, and immune response. The genes were also significantly associated with various brain regions but not with non-brain tissues (see Figure 2C). Particularly, all brain regions available in GTEx were identified with *p*-values less than 0.05, but other tissues were not significant (*p*-values greater than 0.05). This finding is consistent with the results of previous studies in which the authors analyzed genomic data and identified genes and processes associated primarily with the brain [20,23,25,26]. Thus, to reveal more details regarding MDD-related genes, we performed TWAS only for various brain regions.

### 3.2. Genes, Pathways, and Processes Identified by TWAS and Associated with Major Depressive Disorder

A meta-analysis of TWAS results obtained for 15 brain regions identified 371 genes (adjusted *p*-value < 0.05) potentially associated with MDD (see Section 2.2 Results). We suggested that an increased transcription of 187 genes and decreased transcription of 184 genes were associated with increased MDD risk. Two hundred sixty-nine out of the 371 genes (72.5%) were novel, as they were not revealed in previous TWAS studies [16,17,18,20,22,23,26] (see Table S8). We compared the obtained genes with genes identified by colocalization analysis and Mendelian randomization (see Section 2.2 Results). According to colocalization results, 35 of 371 associations with MDD are driven by shared causal SNPs (see Table S6). Mendelian randomization identified 94 of 371 genes whose transcription changes may have a causal effect on the development of MDD. According to HEIDI analysis, the expression of 67 of 94 genes and the MDD are potentially affected by the same underlying causal SNPs (see Table S7). The genes identified by TWAS and supported by results of colocalization analysis and Mendelian randomization are the most probable candidates for a role in the etiopathogenesis of MDD. However, the percentage of 371 genes supported by colocalization analysis (35 out of 371 genes, 9.4%) and Mendelian randomization (94 out of 371 genes, 25.3%) is relatively low. We propose that this may be explained by the small number of samples in the eQTL studies (from hundreds to thousands) compared to GWAS (hundreds of thousands of samples), which makes it difficult to identify a large number of common SNPs.

Enrichment analysis identified signaling and metabolic pathways and cellular processes associated with MDD (Figure 3 and Figure 4). We manually estimated the positions of 371 genes in the pathway maps, and their functions described in the UniProt database allowed us to classify genes and pathways into functional categories (see Table 1, Table 2 and Table 3). In addition, several genes involved in enriched pathways and identified in at least three brain regions with an unadjusted *p*-value < 0.05 were selected. This approach allowed the identification of additional MDD-related genes, whereas focusing on only genes from enriched pathways potentially reduced the number of false positive results.

Four main groups of MDD-related genes were revealed: genes participating in metabolic and signaling pathways and genes participating in various cellular processes and gene expression processes. In addition, genes can be classified as involved in neurotransmitter metabolism, transport, and release, and genes involved in other cellular processes.

#### 3.2.1. Genes and Processes Associated with Brain Metabolism

Various enzymes involved in the metabolism of two neurotransmitters, glutamate and glycine, were identified by TWAS. Analysis of the predicted gene expression changes and gene functions allowed us to propose the increase in glutamate and glycine levels that, in turn, may predispose to MDD. For instance, the increase in transcription of the FOLH1 gene encoding glutamate carboxypeptidase II was predicted to be associated with the risk of MDD [78]. Glutamate carboxypeptidase II is known to modulate excitatory neurotransmission in the brain through the hydrolysis of the neuropeptide N-acetylaspartylglutamate (NAAG), thereby releasing glutamate. The decreased level of solute carrier family 1 members 6 and 7 (SLC1A6, SLC1A7) was also predicted to be associated with MDD. These transporters are responsible for the uptake of L-glutamate in synapses and play roles in terminating the postsynaptic action of glutamate. The decreased expression of dihydrolipoamide dehydrogenase (DLD) and aminomethyltransferase (AMT) was predicted by TWAS to be associated with the risk of MDD. These enzymes are the components of the glycine cleavage system, and their decrease may increase the glycine level in the brain. The role of glycine and glutamate in MDD is discussed in the literature. Most studies showed that the decreased level of glutamate was associated with MDD, whereas a few studies showed that an increased glutamate level can also be associated with MDD [79,80,81]. The role of glycine in MDD development is less studied. Most of the studies investigated the role of glycine as a co-agonist of glutamatergic NMDA receptors [82,83,84,85,86]. It was shown that a decreased glutamine level in plasma correlated with the severity of MDD and that glycine increased glutamatergic signaling with an antidepressive effect. This finding is contrary to the TWAS results. However, the predicted increase in glycine level agrees with the predicted increase in glutamate level because glycine is a co-agonist of NMDA receptors. Thus, the increase in these neurotransmitters may potentially increase MDD risk in at least part of the individuals. Additionally, we revealed other receptors for neurotransmitters, including serotonin, dopamine, and cholinergic receptors whose relationship with MDD is well known.

Besides neurotransmitter metabolism and transport, other processes with less clear links to MDD were identified. The revealed processes are apparently not specific to MDD and may be associated with other psychiatric and neurological diseases. Various genes, pathways, and processes related to brain energy metabolism were predicted to be decreased in MDD (see Figure 3 and Table 1). They include processes of glycolysis, fatty acid metabolism, the tricarboxylic acid cycle, and oxidative phosphorylation. The decrease in brain metabolism in MDD was confirmed by various experimental findings [50]. The decline in brain metabolism disrupts various processes, including axonal action potentials, cell signaling, presynaptic Ca^2+^ entry, the uptake and recycling of neurotransmitters, and synaptic vesicle release, that may contribute to MDD development.

Changes in lipid metabolism are another mechanism of MDD development [53]. We identified various genes and pathways related to the metabolism of unsaturated fatty acids and phospholipids. The associations between lipid metabolism and MDD are extensively described in the literature [53,55,87].

The largest number of revealed enzymes were related to glycan biosynthesis, including N- and O-glycans and glycosaminoglycans, as well as to the synthesis of GPI-anchors. Glycans are essential to cell–cell and cell–ECM interactions, including those in neural systems [62,88,89,90,91,92,93].

#### 3.2.2. Genes and Processes Associated with Gene Expression and Cellular Functions

The large number of genes encode proteins involved in DNA repair (see Table 2). The decrease in transcription of these genes, and thus the decrease in DNA repair processes, was predicted to be associated with MDD risk. This finding is supported by the large number of experimental observations described in the literature. The DNA damage observed in MDD may mainly be caused by oxidative stress, which has been hypothesized to constitute a pathogenic mechanism in depression. The combination of DNA damage with reduced DNA repair capacity may cause an accumulation of unrepaired damaged DNA, ultimate neuronal loss, and associated neural deterioration, and, thus, contribute to the development of MDD or other psychiatric diseases [63,64].

Gene expression processes, from the regulation of transcription to protein maturation and transport in the cell, were predicted to be associated with MDD. Chromatin remodeling is an important mechanism of gene transcription regulation. We predicted that the increase in transcription of genes encoding components of the INO80 complex and Polycomb group proteins may be associated with the risk of MDD. On the contrary, the transcription of the H1.2 linker histone (H1-2) and the H2A.Z variant histone 2 (H2AZ2) genes was predicted to be decreased. The INO80 complex is an ATP-dependent nucleosome remodeler that is implicated in a diverse array of functions, including transcriptional regulation, DNA replication, and DNA repair [94]. In particular, it modulates vital steps in cortical neurogenesis [95]. H2A.Z is a variant histone which replaces conventional H2A in a subset of nucleosomes and is involved in processes of transcription regulation, DNA repair, DNA replication, and chromosomal stability. It plays a crucial role in neurodevelopment and brain functions [96], including mitochondrial function, neuron survival, cortical neurogenesis, learning, and memory [97,98,99]. Its depletion can disrupt these processes. Interestingly, the INO80 complex, which was predicted to increase in MDD by TWAS, replaces H2A.Z in nucleosomes with the canonical H2A histone [100,101]. Along with chromatin remodelers, we identified enzymes performing the post-translational modification of histones: lysine methyltransferase 5A (KMT5A), protein arginine methyltransferase 6 (PRMT6), and SET domain bifurcated histone lysine methyltransferase 2 (SETDB2). The role of histone modifications in depression is also described in the literature [66].

We found several proteins involved in the processing of mRNA, tRNA, and rRNA, including polyadenylation and the modification of nucleotides. For example, FAST kinase domain 5 (FASTKD5) and mitochondrial poly(A) polymerase (MTPAP) are involved in mitochondrial mRNA processing, and mitochondrial rRNA methyltransferase 2 (MRM2) is involved in the biogenesis of a large subunit of the mitochondrial ribosome [102]. The role of these proteins in MDD is not clear. It was only shown that the THUMP domain containing 3 (THUMPD3) and exoribonuclease 1 (ERI1) associated with genetic variations contributed to both MDD and type 2 diabetes [103]. We identified various genes encoding proteins participating in RNA splicing. We proposed that the SNP-induced changes in expression of these genes may influence alternative splicing. The alternative splicing of mRNA is a major mechanism by which the proteomic diversity of eukaryotic genomes is amplified. It is known that the abnormalities of splicing may influence various processes in the brain: cell-fate determination, axon guidance, synaptogenesis, synaptic vesicle release, neurotransmitter release, and others. Thus, the abnormalities of splicing can be related to the development of various neuropsychiatric disorders [65]. There is limited evidence on the role of particular splicing-related proteins in depression and related conditions. For instance, the relationship between the increase in the transcription of RNA binding motif protein 8A (RBM8A) and the risk of MDD was predicted by TWAS. Alachkar and colleagues showed that the overexpression of RBM8a in the mouse dentate gyrus leads to increased anxiety-like behavior, abnormal social interaction, and decreased immobile time in the forced swimming test [104].

The transcription of various genes involved in the regulation of translation was predicted to be increased in MDD. They include genes encoding ribosomal proteins, regulatory proteins, and tRNA synthetases (Table 2). Generally, a decrease in protein translation is associated with MDD [67]. However, recently, Vandana Sharma and colleagues proposed a “ribosome hypothesis” of mood disorders, including MDD [68]. According to the hypothesis, besides global changes in protein synthesis, local alterations in ribosome composition may occur and lead to ribosome specialization that affects synaptic function in a compartment-specific manner. Specialized ribosomes are a subset of heterogeneous ribosomes that selectively modulate the translation of specific mRNAs. Thus, the up-regulation of particular ribosomal proteins may change the synthesis of particular proteins involved in MDD. The same is potentially true for other translation regulatory proteins revealed by TWAS. Interestingly, most of the revealed proteins are involved in protein translation in the mitochondria: mitochondrial ribosomal protein L34 (MRPL34), GTP binding elongation factor GUF1 (GUF1), mitochondrial translation release factor 1 like (MTRF1L), tRNA synthetase 2, mitochondrial (VARS2), and histidyl-tRNA synthetase 2, mitochondrial (HARS2). This may suggest an increase in mitochondrial protein synthesis in MDD.

The same principle may be true for the revealed changes in vesicular transport. We identified several components of the SNARE complex (BNIP1, SCFD1, STX17, GOSR2, VAMP2) (see Table 2) involved in synaptic vesicular fusion and the release of neurotransmitters. The transcription of the corresponding genes was positively associated with the risk of MDD by TWAS. It was shown that the expression of VAMP2 and other genes of the SNARE complex was increased in the hippocampus and prefrontal cortex of rats that underwent prenatal stress. Increased SNARE proteins and SNARE complex formation, in turn, cause an increase in glutamate and its downstream excitotoxicity, contributing to depression [105].

We identified that the risk of MDD was associated with the increase in transcription of genes related to the unfolded protein response and cell death and with the decrease in transcription of genes related to the cell cycle (Table 2). In particular, gasdermins mediate pyroptosis, which is an inflammatory cell death process with links to neuroinflammation and is closely associated with depression [106]. The expression of cell cycle regulators is necessary for adult neurogenesis, neuronal migration, dendrite morphogenesis, synaptic maturation, and plasticity [71].

TWAS predicted a decrease in transcription of genes encoding cytoskeletal proteins such as actin regulators and motor proteins such as myosins, kinesins, and dyneins (see Table 2). These proteins participate in the processes of the anterograde and retrograde transport of vesicles with neurotransmitters and proteins in neurons. Axonal transport is a critical mechanism for normal neuronal function, playing crucial roles in axon growth, neurotransmitter secretion, normal mitochondrial function, and neural survival. It was shown that stress-induced depression in rats was associated with the down-regulation of kinesin and dynein genes [72].

Several genes encoding proteins participating in cell adhesion to the ECM, including integrins and protocadherins, were also identified (see Table 2). The degradation or remodeling of the perivascular and perineuronal system induced by injury or stress contributes to various neurological and psychiatric diseases, including depression [107]. The dysregulation of the local balance between extracellular proteases and the ECM activates multiple temporally overlapping signaling cascades, resulting in impaired synaptic plasticity and cognition. The deviation in each direction affects synaptic plasticity and tissue regeneration both in beneficial and detrimental ways, depending on the involved molecules. In particular, TWAS identified both the decrease and increase in transcription of protocadherins to be related to MDD, depending on particular genes: the decrease in protocadherins alpha 7, 8, and 10 (PCDHA7, PCDHA8, and PCDHA10, respectively) and the increase in protocadherin alpha 13 (PCDHA13) and protocadherins beta 2, 3, 8, and 10 (PCDHB2, PCDHB3, PCDHB8, and PCDHB10, respectively). These protocadherins are involved in cell adhesion, neurite initiation and outgrowth, axon pathfinding and fasciculation, and synapse formation and stabilization. The tissue distribution and function of particular protocadherins are different [76]. Thus, the changes in content of particular protocadherins may change these processes and contribute to diseases, including MDD.

#### 3.2.3. Genes Involved in Signaling Pathways

We identified several signaling pathways as related to MDD and more thoroughly estimated the role of receptors and their ligands because they are extremely important for communication between neurons and other cells. Besides receptors for the neurotransmitters described above, we identified two large groups of receptors and ligands: hormones and growth factors, and immune proteins. The role of central and peripheral inflammation in the pathogenesis of MDD is well described in the literature [70]. What is less trivial are the links with MDD for anti-Mullerian hormone (AMH), growth differentiation factor 9 (GDF9), and nuclear receptor subfamily 1 group H member 3 (liver X receptor alpha) (NR1H3). Anti-Mullerian hormone and growth differentiation factor 9 are involved in the regulation of ovarian folliculogenesis. The transcription of genes encoding both hormones was predicted by TWAS to be increased. However, to date, no associations were found for anti-Mullerian hormone level and depression [108,109], whereas growth differentiation factor 9 was not investigated in relation to MDD. Liver X receptor alpha is expressed in the brain and acts as a central cholesterol regulator, is involved in myelination and remyelination processes, maintains the blood–brain barrier, provides neuroprotective effects, and lowers neuroinflammation [110,111,112].

#### 3.2.4. Genes Involved in Immune Response and Inflammation

We identified several immune and inflammation-related genes that play an important role in the etiopathogenesis of MDD (see Table 3). Since the analysis was performed only on brain regions, several important immune genes may be missed; therefore, we performed TWAS for whole blood data available in GTEx. We did not reveal additional immune-related genes that were not identified by TWAS analysis of brain regions (see Table S12). Additional immune-related genes associated with MDD could potentially be identified in future studies using large eQTL datasets for specific immune cell types.

### 3.3. The Role of Identified Genes and Processes in Other Diseases

The genes and processes identified by TWAS do not appear to be specific to MDD but rather are associated with several psychiatric disorders. In particular, bipolar disorder and schizophrenia shared many molecular mechanisms with MDD. For instance, all three disorders are associated with altered energy metabolism [50], increased DNA damage due to oxidative stress [63,64], dysfunctional gene splicing [65], and impaired mRNA translation [67]. Various processes linked to MDD by an analysis of TWAS results are known to be associated with other, non-psychiatric disorders. For instance, several processes are common between MDD and cancer. In particular, the deregulation of DNA repair pathways is associated with the initiation and progression of cancer [113,114] and may contribute to the development of MDD [63,64]. TWAS identified various chromatin components and regulators, including histones, components of the INO80 complex, Polycomb group proteins, and enzymes performing the post-translational modification of histones (see Section 3.2.2). The role of these genes and corresponding proteins in the development of MDD is supported by literature data [66,94,95,96,97,98,99,100,101]. In particular, transcription of genes encoding INO80 complex and Polycomb group proteins was predicted to be increased in MDD (see Table 2). The overexpression or aberrant expression of the INO80 complex and Polycomb group proteins leads to uncontrolled cell growth, invasion, and resistance to cell death. Thus, these proteins are also clearly associated with cancer development [115,116,117,118]. The lysine methyltransferase 5A (KMT5A) was also predicted to be increased in MDD. KMT5A is known to promote cancer cell proliferation, motility, migration, invasion, metastasis, and drug resistance, and its inhibitors can be used in cancer therapy [119]. For more information on the relationship between depression and cancer, see review [120].

### 3.4. Comparison of Genes and Pathways Obtained by TWAS with Genes and Pathways Identified by Differential Expression Analysis in Post-Mortem Brain Regions

To validate transcriptional changes predicted by TWAS, we identified DEGs in post-mortem brain regions between individuals with and without MDD. We identified a relatively low number of DEGs that agrees with the results of other authors. It was shown that the number of DEGs is lower in MDD than in bipolar disorder and, especially, in schizophrenia [32,36]. This may be a feature of MDD, but it can also be explained by the features of the data. Brain-derived samples may be heterogenic due to individual differences in transcription. The datasets used in the study (GSE80655, GSE101521, GSE102556) contain transcription data obtained from subjects with and without MDD. All subjects were group-matched for age, pH and postmortem intervals (PMI). Moreover, datasets include only subjects who died suddenly, to avoid metabolic complications related to agonal effects. In addition, we accounted for confounding factors presented in the datasets by including them as covariates in the statistical models. These factors included age of subjects, PMI, pH, RNA integrity number (RIN), alcohol use, and medication use depending on the dataset. Thus, the transcription profiles obtained are probably free from significant influence of confounding factors. Moreover, the analysis of all three datasets showed the same results (Figure 5, Figure 6, Figure 7 and Figure 9), which indicates the reliability of the obtained results.

Previous studies have shown the sex-specific transcriptional changes in MDD. Thus, we performed transcriptional analysis for males and females separately. We found few DEGs that intersected with TWAS-derived genes, whereas the intersection at the level of pathways was much greater (see Figure 5, Figure 6 and Figure 7 and Table S10). Nevertheless, many pathways identified by analysis of the TWAS results were not confirmed at the transcriptomic level. Several hypotheses can be suggested to explain this disagreement. First, TWAS was performed based on the results of GWAS that included hundreds of thousands of individuals and took into account rare alleles, whereas transcriptomic studies were performed based on a few samples of the brain (from 3 to 19 samples; see Table 4). Thus, transcriptional changes that occur in a few individuals cannot be identified by transcriptomic analysis. Second, MDD-related SNPs, genes, and pathways are associated with the predisposition to MDD, whereas transcriptional changes were revealed in post-mortem brains from individuals that were already ill. The corresponding mechanisms may be different. Third, we found that some genes and pathways were differentially regulated in either males or females, whereas some were changed in opposite directions. The direction of transcriptional changes predicted by TWAS was similar to transcriptional changes observed in either males or females depending on the gene and pathway. This may be explained by the particular features of eQTL data that were used by TWAS. This was obtained using transcription profiles from males and females together. To identify sex-specific TWAS-derived genes, it is necessary to perform new large GWASs for males and females separately.

The investigation of differences in transcriptional changes between males and females is extremely important because of the different treatment of MDD potentially required. There are several studies that focus on sex-specific MDD mechanisms, including transcriptomics studies [31,34,40,41,42,121]. Thus, we focused on the intersection between TWAS and transcriptomic results in our study rather than sex-specific differences themselves.

First, we found some genes and pathways that were changed in opposite directions. For example, complement components C4A and C4B were up-regulated in females but down-regulated in males, whereas TWAS showed an increase in C4A and a decrease in C4B gene transcription (see Figure 5). Complement is known to be involved in neuroinflammation and synaptic plasticity [122,123,124], and it was shown that overexpression of C4A in mice reduced cortical synapse density, increased the microglial engulfment of synapses, and altered mouse behavior [123]. The plasma level of C4A in a mixed group of males and females with MDD was significantly increased [124]. The pathway related to glycolysis was up-regulated in females but down-regulated in males, in contrast to the oxidative phosphorylation pathway, which was up-regulated in males but had a mixed pattern of activity in females depending on the brain region (Figure 6). The pathway “protein processing in endoplasmic reticulum” was up-regulated in most brain regions in females and down-regulated in males (Figure 7 and Figure 8). All these pathways were predicted as down-regulated by TWAS. The direction of gene expression changes predicted for genes from the pathways by TWAS was more similar to the observed transcription changes in males than to those observed in females. Thus, to identify sex-specific genomic SNPs, genes, and pathways, new large GWASs performed separately for males and females are required.

Second, we identified genes and pathways whose expression and activity were changed in either females or males. Interestingly, in general, females exhibited more up-regulated genes and pathways, whereas males showed more down-regulated genes and pathways (Figure 5, Figure 6 and Figure 7).

Third, most of the pathways that were identified by both TWAS and transcriptomic analysis belonged to signaling pathways, whereas there were much fewer corresponding intersections for metabolic pathways and pathways describing gene expression (Figure 6 and Figure 7).

### 3.5. Master Regulators Potentially Responsible for Observed Gene Expression Changes in Major Depressive Disorder

In the final stage of our study, we identified MRs, which are the proteins in the signaling network potentially responsible for the change in gene expression revealed by both TWAS and transcriptomic analysis (see Section 2 and Table S11). They contribute to both types of MDD mechanisms: at the levels of SNP-induced gene expression changes and transcriptional changes in the brains of individuals with MDD. We identified “active” MRs, which activate the transcription of up-regulated genes and repress the transcription of down-regulated genes, and “inactive” MRs, which repress the transcription of up-regulated genes and activate the transcription of down-regulated genes. If “inactive” MRs were “active”, the transcription profile would change to a “normal” healthy state [125,126,127]. In our study, we focused on receptors and ligands predicted as MRs, since they are extremely important for communication between cells in the brain (Figure 9). We found that the revealed MRs mainly belong to proteins participating in neural development, differentiation and functions, sex hormones, and immune proteins. Importantly, the “activity” of some of them was opposite in males and females. The revealed MRs were generally “active” in females but “inactive” in males. The MRs predicted based on TWAS results were generally “active”. In particular, we found several sex-related hormones as MRs: anti-Mullerian hormone (AMH), growth differentiation factor 9 (GDF9), and gonadotropin releasing hormone 1 (GNRH1). The transcription of genes encoding these hormones was predicted by TWAS to be increased, and they were predicted as “active” MRs. Growth differentiation factor 9 was also revealed as MR at the level of the transcriptome, but its activity was dependent on sex: “active” MR in females and “inactive” MR in males. Growth differentiation factor 7 (GDF7) and semaphorin 7A (SEMA7A) have a similar pattern of “activity” (see Figure 9). There is no information regarding the role of these proteins in MDD. It is only known that semaphorin 7A is involved in neuroglial remodeling [128], neurogenesis [129], synapse elimination in the developing mouse brain [130], and neuroinflammation [131,132,133]. Interestingly, semaphorin 7A expression is regulated during the estrous cycle by the fluctuating levels of gonadal steroids [128]. The opposite “activity” in males and females was also revealed for frizzled class receptors 4 and 7 (FZD4 and FZD7), which are part of the Wnt signaling pathways. The Wnt pathway is essential for neurodevelopment and is a downstream target of antidepressants with a potential relationship with MDD [134].

The identified MRs may partially explain sex differences in the mechanisms of MDD development and the increased susceptibility of females to depression. Some of the receptors and ligands are clearly linked to neural development, differentiation, and functions, such as growth differentiation factor 7 (GDF7), neogenin 1 (NEO1), neurotrophic receptor tyrosine kinase 1 (NTRK1), and semaphorin 7A (SEMA7A). Another group of MRs includes hormones involved in gonad differentiation: anti-Mullerian hormone (AMH), growth differentiation factor 9 (GDF9), and gonadotropin releasing hormone 1 (GNRH1). Thus, the sex differences in MDD can be driven by differences in sex hormones’ influence on brain developmental processes and functions of the adult brain [41]. It is known that the changes in ovarian hormones during the different stages of the menstrual cycle, postpartum period, or menopause are associated with the onset of depression. Sex hormones influence both the brain’s sexual differentiation and depression susceptibility [135].

Since MRs are some of the key proteins regulating gene expression and cell/tissue/organ functions, they can be considered potential therapeutic targets. Many of the MRs identified in this study were encoded by genes revealed by TWAS or transcriptomic analysis, increasing the probability of their role in MDD and their success as potential drug targets. They can potentially regulate various processes revealed as related to MDD, from neurotransmitter biosynthesis to DNA repair (see above). The pharmacological perturbation of these processes by drug action on MRs may induce an improvement in MDD. It was shown that the co-administration of existing antidepressants with anti-inflammatory drugs [136,137] increases the therapeutic effect. It seems to also be possible to prevent or reverse psychiatric disorders, including MDD, by preventing DNA damage [64]. However, it should be noted that the sex-specific “activity” of MRs means that the treatment of MDD must also be sex-specific. Moreover, the pathological processes identified in the study may not be present in all patients. For instance, inflammation is present and potentially contributes to the development of MDD in only a subset of patients, so anti-inflammatory therapy may only be effective in the corresponding subgroup [6]. Thus, effective treatment of MDD requires the discovery and application of biomarkers that stratify patients based on various genes and processes, including neuroinflammation, decreased DNA repair capacity, changes in regulation of gene expression, etc.

## 4. Materials and Methods

### 4.1. Mapping Single-Nucleotide Polymorphisms Associated with Major Depressive Disorder to Human Genes

To identify SNPs and associated genes that are related to MDD, summary statistics from the meta-analysis by Howard and colleagues were used [19]. The corresponding data was obtained from the Psychiatric Genomics Consortium (https://pgc.unc.edu/, accessed on 23 March 2024) and included 8,483,301 SNPs with information on alternative and reference alleles, *p*-values, and odds ratios. The 4625 SNPs were selected for analysis according to the most frequently used *p*-value threshold of 5 × 10^−8^ [138]. These SNPs were mapped to human genes using Variant Effect Predictor (VEP) [44] (https://www.ensembl.org/info/docs/tools/vep/index.html, accessed on 23 March 2024) and Functional Mapping and Annotation of Genome-Wide Association Studies (FUMA GWAS) [43] (https://fuma.ctglab.nl/, accessed on 27 May 2024). To estimate the functional significance of missense variants, SIFT and PolyPhen predictions in VEP output were used. The missense variant was considered significant when at least one of two algorithms predicted the variant to be deleterious.

MAGMA software (version 1.08) was used, as part of the FUMA platform, to identify genes and gene sets associated with MDD. For each gene, the *p*-values of all polymorphisms mapped to the gene are aggregated to calculate a single *p*-value. We considered all polymorphisms to be localized within the gene body and at a distance of 10 thousand base pairs beyond it, in accordance with the recommendations of the authors of the algorithm [45]. Similar analysis was performed to identify pathways and tissues enriched by the revealed genes using the obtained gene-level statistics. This analysis revealed KEGG (https://www.genome.jp/kegg/pathway.html, accessed on 27 May 2024) and Reactome (https://reactome.org/, accessed on 27 May 2024) pathways, Gene Ontology biological processes (https://geneontology.org/, accessed on 27 May 2024), and GTEx v8 (https://www.gtexportal.org/home/, accessed on 27 May 2024) organs and tissues associated with MDD.

### 4.2. Transcriptome-Wide Association Study

To identify gene transcription changes caused by SNPs and associated with increased MDD risk, we performed TWAS based on eQTL for several brain regions. The analysis was performed using S-PrediXcan [46] (https://github.com/hakyimlab/MetaXcan, accessed on 24 April 2024) software, which requires summary statistics instead of individual-level genomics data. For the analysis, we used whole summary statistics from the Howard study containing 8,483,301 SNPs and summary statistics on eQTL for brain regions that were obtained from the Genotype-Tissue Expression (GTEx) Portal (https://gtexportal.org/, accessed on 27 May 2024). The summary statistics for eQTL include data on SNPs, coefficients from corresponding linear models, and *p*-values. S-PrediXcan merges summary statistics from the GWAS study and eQTL studies and predicts the direction of transcription changes in a particular gene that is associated with an increased risk of disease. We used GTEx eQTL data from the following brain regions: amygdala, anterior cingulate cortex, caudate, cerebellar hemisphere, cerebellum, cortex, frontal cortex, hippocampus, hypothalamus, nucleus accumbens, putamen, spinal cord cervical c-1, and substantia nigra (see Section 2). Additionally, we used eQTL data from two other sources: (1) data on the brain dorsolateral prefrontal cortex from the CommonMind consortium [139], and (2) data on the frontal and temporal cerebral cortex from PsychENCODE [140]. To perform TWAS, we used Elastic Net-based models in S-PrediXcan because their output statistics can be used for pathway enrichment analysis (see below). As a result, gene-level statistics were obtained for each brain region. Statistics include gene identifiers, z-scores, *p*-values, and values of the models’ accuracy. The positive and negative z-scores reflect the direction of gene transcription changes caused by SNPs that are associated with the increased risk of MDD.

To calculate a single *p*-value for each gene, the sum of logs method, also known as Fisher’s method, was used to combine *p*-values obtained for 15 brain regions. We applied the sumlog function from the metap R package (version 1.12) for this purpose.

To identify KEGG and Reactome pathways associated with the revealed genes, Genotype Imputed Gene Set Enrichment Analysis (GIGSEA) [141] was performed, which is similar to Gene Set Enrichment Analysis (GSEA) [142] that is applied to transcriptomic data. GIGSEA identified pathways enriched by genes with top positive or negative z-scores. In contrast to GSEA, which was applied to experimentally determined transcription profiles, GIGSEA performed enrichment analysis based on gene expression predicted by S-PrediXcan, and this prediction had limited accuracy. The uncertainty in prediction is quantified as correlation (r2) between the measured and predicted gene expression from cross-validation by S-PrediXcan. This uncertainty is taken into account as weights in a linear regression model [141]. The corresponding analysis was performed using the GIGSEA R package (version 1.24.0).

### 4.3. Identification of Differentially Expressed Genes and Pathways in Post-Mortem Brain Tissues

The information on gene expression in post-mortem brain tissues from individuals with and without MDD was obtained from Gene Expression Omnibus (GEO) (https://www.ncbi.nlm.nih.gov/geo/) (accessed on 11 April 2025). The dataset GSE80655 was created by Ramaker R.C. and colleagues [36] and included transcription data from the anterior cingulate gyrus, dorsolateral prefrontal cortex, and nucleus accumbens. Controls were selected based on the absence of severe psychiatric disturbance and mental illness within first-degree relatives. To limit the effect of acute patient stress at the time of death as a potential confounder, only patients with an agonal factor score of zero and a minimum brain pH of 6.5 were included in the study. The dataset GSE101521 was created by Pantazatos S.P. and colleagues [35] and included transcription data from the dorsolateral prefrontal cortex. All subjects were selected because they died suddenly to avoid metabolic complications related to agonal effects. All brains were free of gross neuropathology and had negative brain toxicology for psychotropic, illicit psychoactive drugs, and neurotoxic drugs. There were no diagnoses of alcohol or drug use disorders. Antemortem medication history for three months ruled out recent exposure to psychotropic medication and confirmed results of comprehensive peripheral and brain toxicology. The dataset GSE102556 was created by Benoit Labonté and colleagues [31] and included transcription data from the orbitofrontal and dorsolateral prefrontal cortex, cingulate gyrus 25, anterior insula, nucleus accumbens, and subiculum. Subjects were group-matched for age, pH, and postmortem intervals (PMI). All subjects died suddenly without a prolonged agonal state.

Differential expression analysis was performed using functions from the edgeR R package (version 4.4.2). For instance, the filterByExpr function was used with default parameters to filter out genes with a low expression level before analysis. The calcNormFactors function was used to normalize read counts by taking into account raw library sizes. DEGs were identified using estimateDisp, glmQLFit, and glmQLFTest functions. Analysis was performed for samples from females and males separately. In this stage, we accounted for confounding factors presented in the datasets by including them in the statistical models as covariates. These factors included age for the GSE101521 dataset; age, post-mortem interval (PMI), and pH for the GSE80655 dataset; and age, RNA integrity number (RIN), alcohol use, and medication use for the GSE102556 dataset.

To identify KEGG pathways associated with gene expression changes, we used GSEA implemented in the clusterProfiler R package (version 4.14.4).

### 4.4. Identification of Master Regulators Based on TWAS Results and Transcriptomic Data

Master regulators (MRs) were identified for both TWAS-derived data and initial transcriptomic data. Two types of MRs were identified: transcription factors (TFs) and their upstream regulators, including kinases, phosphatases, receptors, and ligands. At the first step of analysis, TFs were identified, and the obtained information was then used to identify upstream MRs. Data on the TF–gene interactions required for the analysis was obtained from the CollecTRI database [143] (accessed on 11 April 2025). The GIGSEA algorithm was used to identify TFs for TWAS-derived data [141]. In addition, an algorithm similar to GSEA implemented in the viper R package (version 1.40.0) was used to identify TFs for transcriptomic data [144]. Both GIGSEA and viper algorithms calculate positive and negative scores for each TF. Positive scores mean that the TF activates the transcription of up-regulated genes and represses the transcription of down-regulated genes; therefore, the TF can be considered “active”. By contrast, negative scores mean that the TF represses the transcription of up-regulated genes and activates the transcription of down-regulated genes. This TF can be considered “inactive”; if it were active, the transcription profile would change to a “normal” healthy state.

Upstream MRs were identified by calculating the activating and inhibiting shortest paths in the signaling network from each potential upstream regulator to TFs revealed in the previous step. The signaling network with the edges of two types, activating and inhibiting, was obtained from the OmniPath database [145]. The corresponding data was loaded using functions from the OmnipathR package (version 3.14.0). The identification of upstream MRs was performed using the CausalR package (version 1.38.0) with a maximal length of shortest paths of 5 as the default parameter [146]. This analysis was performed separately for different brain regions using tissue-specific signaling networks. To make the OmniPath network tissue-specific, we removed all nodes corresponding to genes that were not expressed in the analyzed tissue. To make the tissue-specific network in the case of transcriptomic data, we simply included genes presented in the corresponding read counts table after removing genes with low expression (see above). To construct the tissue-specific network in the case of TWAS-derived data, we included genes with tpm (transcripts per million) values more than 1 in the corresponding brain region using consensus tpm values from the Human Protein Atlas (https://www.proteinatlas.org/, accessed on 14 October 2024).

To reduce the number of false positive results, we selected MRs that were differentially expressed based on both TWAS-derived and transcriptomics data [125,126,127] (see Section 2).

## 5. Conclusions

We performed a combined analysis of genomic and transcriptomic data to identify the key genes, pathways, and master regulators involved in the development of major depressive disorder. The most important results are provided below.

A transcriptome-wide association study identified 371 genes associated with major depressive disorder. Two hundred sixty-nine out of 371 genes (72.5%) were novel, as they were not revealed in previous TWAS studies.

We identified that single-nucleotide polymorphisms may change the wide range of processes in the brain that predispose to the development of major depression. In contrast to previous studies, we identified that polymorphisms may contribute to an increase in the level of glycine and glutamate, which may increase the risk of depression development.

Besides changes in the metabolism and transport of neurotransmitters and increases in neuroinflammation, we revealed less trivial processes whose disruption may contribute to the increase in depression risk. These include decreases in energy metabolism in the brain; changes in phospholipid and glycan metabolism; decreases in DNA repair; changes in processes of chromatin remodeling and histone modifications; changes in mRNA, tRNA, and rRNA processing and splicing; the translation of mRNA, especially in the mitochondria; increases in cell death processes and decreases in cell cycles; and processes of cell–cell and cell–extracellular matrix interaction. The role of many of these processes in major depression has not yet been investigated.

The findings obtained at the genome level were confirmed at the transcriptome level. The gene expression changes in the brain related to major depression were mostly sex-specific, and the transcription of many genes was changed in opposite directions in males and females. Generally, an increase in identified processes was observed in females, and a decrease was observed in males. However, we were not able to identify corresponding sex-dependent differences by transcriptome-wide association analysis because of the absence of corresponding genome-level data. Thus, it is necessary to perform new large GWASs for males and females separately.

Master regulators responsible for transcriptional changes in the revealed genes and the modulation of associated processes were identified. We revealed some novel master regulators: anti-Mullerian hormone, growth differentiation factor 9, growth differentiation factor 7, semaphorin 7A, and frizzled class receptors 4 and 7. Most of them were active in females but inactive in males. The revealed genes, processes, and master regulators can be considered potential therapeutic targets for the treatment of major depressive disorder. However, the sex-specific characteristic of their changes in depression requires different therapeutic modulations in males and females. Since the pathological processes identified in the study, such as inflammation or reduced capacity for DNA repair, may not be present in all patients, patient stratification strategies need to be developed and implemented to improve treatment efficacy.

## Figures and Tables

**Table 1:** Metabolic enzymes that were predicted to be associated with major depressive disorder using TWAS. The ↑ and ↓ arrows represent the predicted direction of gene expression changes associated with an increased risk of major depressive disorder. An asterisk * means that the gene has an adjusted *p*-value < 0.05 that is based on a meta-analysis of 15 brain regions. The symbol ^#^ means that the gene was identified by colocalization analysis. The symbol ^&^ means that the gene was identified by Mendelian randomization.

Functional Category	Protein Name	Main Known Function
Fatty acid metabolism	Acyl-CoA synthetase family member 3 (ACSF3 *) ↓	Intramitochondrial fatty acid synthesis
	Fatty acid desaturase 1 (FADS1 *) ↓	Synthesis of highly unsaturated fatty acids, phospholipids, and icosanoids
	Hydroxysteroid 17-beta dehydrogenase 8 (HSD17B8 *) ↓	Fatty acid synthesis
	Sterol carrier protein 2 (SCP2) ↓	Peroxisomal oxidation of branched-chain fatty acids
Glutamate metabolism	Folate hydrolase 1 (FOLH1 *^&^) ↑	Modulates excitatory neurotransmission through the hydrolysis of the neuropeptide, N-aceylaspartylglutamate (NAAG), thereby releasing glutamate
	Gamma-glutamyl hydrolase (GGH *)↑	Hydrolyzes the polyglutamate sidechains of pteroylpolyglutamates
Glycan metabolism	ALG10 alpha-1,2-glucosyltransferase (ALG10) ↑	N-Glycan biosynthesis
	ALG10 alpha-1,2-glucosyltransferase B (ALG10B) ↓	N-Glycan biosynthesis
	ALG3 alpha-1,3- mannosyltransferase (ALG3) ↑	N-Glycan biosynthesis
	Beta-1,3-glucuronyltransferase 2 (B3GAT2) ↓	Glycosaminoglycan biosynthesis
	Beta-1,3-glucuronyltransferase 3 (B3GAT3 *) ↑	Glycosaminoglycan biosynthesis
	Beta 3-glucosyltransferase (B3GLCT *^#^) ↓	Protein O-linked fucosylation
	Core 1 synthase, glycoprotein-N-acetylgalactosamine 3-beta-galactosyltransferase 1 (C1GALT1 *) ↑	Protein O-linked glycosylation
	Carbohydrate sulfotransferase 10 (CHST10) ↓	Proteoglycan biosynthesis
	Phosphatidylinositol glycan anchor biosynthesis class X (PIGX) ↑	GPI-anchor biosynthesis
	Phosphatidylinositol glycan anchor biosynthesis class Z (PIGZ *) ↑	GPI-anchor biosynthesis
	ST8 alpha-N-acetyl-neuraminide alpha-2,8-sialyltransferase 1 (ST8SIA1) ↑	Glycosphingolipid biosynthesis
	Uronyl 2-sulfotransferase (UST) ↓	Glycosaminoglycan biosynthesis
Glycin metabolism	Dihydrolipoamide dehydrogenase (DLD *) ↓	Component of the glycine cleavage system, acetyl-CoA biosynthesis from pyruvate
	Aminomethyltransferase (AMT *^#&^) ↓	Component of the glycine cleavage system
	Betaine--homocysteine S-methyltransferase (BHMT *) ↓	Converts betaine and homocysteine to dimethylglycine and methionine, irreversible oxidation of choline
	Dimethylglycine dehydrogenase (DMGDH) ↓	Catalyzes the demethylation of N, N-dimethylglycine to sarcosine
	Serine hydroxymethyltransferase 1 (SHMT1 *) ↑	Interconversion of serine and glycine
Hypotaurine metabolism	2-aminoethanethiol dioxygenase (ADO *^&^) ↓	Hypotaurine synthesis
Inositol phosphate metabolism	Adenosine monophosphate deaminase 3 (AMPD3 *) ↑	IMP biosynthesis from AMP
	Inosine triphosphatase (ITPA *) ↓	Hydrolyzes the inosine triphosphate (ITP), deoxyinosine triphosphate (dITP), and xanthosine triphosphate (XTP) to their monophosphate derivatives
Kynurenine metabolism	Aldehyde dehydrogenase 8 family member A1 (ALDH8A1 *) ↑	L-kynurenine catabolic process
Mitochondrial regulation	Inorganic pyrophosphatase 2 (PPA2 *) ↓	Regulation of mitochondrial membrane potential and mitochondrial organization and function
Phospholipid metabolism	Arylsulfatase A (ARSA *) ↓	Hydrolyzes cerebroside sulfate
	Choline kinase beta (CHKB *) ↓	Phosphatidylethanolamine and phosphatidylcholine biosynthesis
	Phospholipase A2 group IVB (PLA2G4B *) ↑	Remodeling of membrane phospholipids
	Sphingomyelin phosphodiesterase 2 (SMPD2 *) ↑	Hydrolysis of sphingomyelin to form ceramide and phosphocholine
Prostaglandin metabolism	Prostaglandin E synthase 2 (PTGES2) ↑	Conversion of prostaglandin H2 into the more stable prostaglandin E2
Tricarboxylic acid cycle	Dihydrolipoamide S-succinyltransferase (DLST) ↑	Conversion of 2-oxoglutarate to succinyl-CoA and CO_2_

**Table 2:** Cellular processes and corresponding genes that were predicted to be associated with major depressive disorder using TWAS. The “Direction” column represents the direction of gene transcription changes induced by SNPs that are associated with an increased risk of major depressive disorder. An asterisk * means that the gene has an adjusted *p*-value < 0.05 that is based on a meta-analysis of 15 brain regions. The symbol ^#^ means that the gene was identified by colocalization analysis. The symbol ^&^ means that the gene was identified by Mendelian randomization.

Process	Direction	Proteins (Genes)
DNA replication and repair	Increase	DEAD/H-box helicase 11 (DDX11 *), PIF1 5′-to-3′ DNA helicase (PIF1 *), Nibrin (NBN), RAD54 like (RAD54L), TIMELESS interacting protein (TIPIN *)
	Decrease	DNA polymerase epsilon (POLE *), DNA polymerase iota (POLI *), DNA topoisomerase III alpha (TOP3A*), O-6-methylguanine-DNA methyltransferase (MGMT *), Nei-like DNA glycosylase 2 (NEIL2 *^&^), PWWP domain containing 3A, DNA repair factor (PWWP3A *), RecQ mediated genome instability 2 (RMI2 *), replication protein A3 (RPA3 *)
mRNA processing	Increase	FAST kinase domains 5 (FASTKD5 *), mitochondrial poly(A) polymerase (MTPAP), exoribonuclease 1 (ERI1)
rRNA processing	Increase	mitochondrial rRNA methyltransferase 2 (MRM2 *)
	Decrease	KRR1 small subunit processome component homolog (KRR1 *), WD repeat domain 55 (WDR55 *)
tRNA processing	Increase	THUMP domain containing 3 (THUMPD3 *^&^), tRNA methyltransferase 61A (TRMT61A *^&^), tRNA phosphotransferase 1 (TRPT1 *), WD repeat domain 6 (WDR6 *)
mRNA splicing	Increase	CASC3 exon junction complex subunit (CASC3), RNA binding motif protein 8A (RBM8A), splicing factor 3b subunit 1 (SF3B1), gem nuclear organelle associated protein 7 (GEMIN7 *), arginine and serine rich coiled-coil 1 (RSRC1 *)
	Decrease	SLU7 homolog, splicing factor (SLU7), small nuclear ribonucleoprotein U4/U6.U5 subunit 27 (SNRNP27), U2 small nuclear RNA auxiliary factor 1 like 4 (U2AF1L4 *), zinc finger matrin-type 2 (ZMAT2 *)
Chromatin regulators	Increase	***INO80 complex***: actin-related protein 5 (ACTR5), INO80 complex subunits E and D (INO80E *, INO80D); ***Polycomb group proteins***: L3MBTL histone methyl-lysine binding protein 2 (L3MBTL2 *), WD repeat domain 5B (WDR5B *); ***histones and modifiers***: lysine methyltransferase 5A (KMT5A)
	Decrease	***Polycomb group proteins***: Scm-like with four MBT domains 1 (SFMBT1); ***histones and modifiers***: H1.2 linker histone (H1-2), H2A.Z variant histone 2 (H2AZ2), protein arginine methyltransferase 6 (PRMT6 *^&^), SET domain bifurcated histone lysine methyltransferase 2 (SETDB2)
Ribosomal proteins	Increase	Mitochondrial ribosomal protein L34 (MRPL34 *), ribosomal protein L12 (RPL12 *), ribosomal protein L36a-like (RPL36AL *)
Translation	Increase	GTP binding elongation factor GUF1 (GUF1 *), HBS1-like translational GTPase (HBS1L *), La ribonucleoprotein 6, translational regulator (LARP6 *), histidyl-tRNA synthetase 1 (HARS1), valyl-tRNA synthetase 2, mitochondrial (VARS2 *^#&^)
	Decrease	Mitochondrial translation release factor 1-like (MTRF1L *), histidyl-tRNA synthetase 2, mitochondrial (HARS2)
Vesicular transport	Increase	BCL2 interacting protein 1 (BNIP1 *), sec1 family domain containing 1 (SCFD1), syntaxin-17 (STX17), Golgi SNAP receptor complex member 2 (GOSR2), vesicle-associated membrane protein 2 (VAMP2 *), dynamin 1 (DNM1), N-ethylmaleimide sensitive factor, vesicle-fusing ATPase (NSF)
	Decrease	B cell receptor associated protein 29 (BCAP29), KDEL endoplasmic reticulum protein retention receptor 2 (KDELR2)
Unfolded protein response	Increase	Activating transcription factor 6 beta (ATF6B *^#&^), glutamine rich 1 (QRICH1 *^#^)
Cell death	Increase	Gasdermins D and E (GSDMD *, GSDME *^&^)
Cell cycle	Increase	EMAP-like 3 (EML3 *), zwilch kinetochore protein (ZWILCH)
	Decrease	Cell division cycle 25B (CDC25B), minichromosome maintenance complex component 6 (MCM6), checkpoint with forkhead and ring finger domains (CHFR *), HAUS augmin-like complex subunit 4 (HAUS4 *), small kinetochore-associated protein (KNSTRN *)
Cytoskeleton	Increase	***Kinesins***: kinesin family member C2 (KIFC2), kinesin light chain 1 (KLC1); ***myosins*:** myosin XVA and XVB (MYO15A *, MYO15B); ***dyneins*:** dynein cytoplasmic 2 light intermediate chain 1 (DYNC2LI1)
	Decrease	***Actin regulators***: actin-related protein 2/3 complex subunit 5-like protein (ARPC5L), diaphanous-related formin 3 (DIAPH3), vinculin (VCL); ***dyneins*:** dynein axonemal heavy chain 7 (DNAH7), dynein cytoplasmic 1 intermediate chain 2 (DYNC1I2), dynein light chain Tctex-type family member 5 (DYNLT5 *); ***kinesins*:** kinesin family member 6 (KIF6)
Cell adhesion	Increase	***Integrins***: integrin binding sialoprotein (IBSP), integrin subunit alpha V (ITGAV *); ***protocadherins***: protocadherin alpha 13 (PCDHA13), protocadherins beta 2, 3, 8, and 10 (PCDHB2 *, PCDHB3, PCDHB8 *, PCDHB10); FRAS1 related extracellular matrix 1 (FREM1 *), collagen type XXVIII alpha 1 chain (COL28A1 *), mucin 4, cell surface associated (MUC4), neuronal growth regulator 1 (NEGR1 *^#&^), neurotrimin (NTM *)
	Decrease	***Integrins***: integrin subunit beta 6 (ITGB6); ***protocadherins***: protocadherins alpha 7, 8, and 10 (PCDHA7 *, PCDHA8 *^&^, PCDHA10 *); cell adhesion molecule 2 (CADM2 *), neurocan (NCAN), peripherin 2 (PRPH2 *)

**Table 3:** Ion channels, receptors, and their ligands from signaling pathways which were predicted to be associated with major depressive disorder using TWAS. The ↑ and ↓ arrows represent the predicted direction of gene expression changes associated with an increased risk of major depressive disorder. An asterisk * means that the gene has an adjusted *p*-value < 0.05, which is based on a meta-analysis of 15 brain regions. The symbol ^#^ means that the gene was identified by colocalization analysis. The symbol ^&^ means that the gene was identified by Mendelian randomization.

Functional Category	Protein Name	Main Known Function
Calcium signaling	Adenylate cyclase 3 (ADCY3) ↓	cAMP synthesis
	Mucolipin TRP cation channel 2 and 3 (MCOLN2, MCOLN3) ↓	Ca^2+^-permeable cation channels
	Nitric oxide synthase 1 and 2 (NOS1, NOS2 *) ↑	Produce nitric oxide (NO). In the brain, NO displays many properties of a neurotransmitter
	Ryanodine receptor 1 (RYR1) ↑	Mediates the release of Ca^2+^ from the sarcoplasmic reticulum into the cytosol
Hormones and growth factors	Anti-Mullerian hormone (AMH *) ↑	Gonadal differentiation, ovarian follicle development
	EPH receptor B2 (EPHB2 *^&^) ↓	Functions in axon guidance during development. Dendritic spine development, formation of excitatory synapses
	Fms-related receptor tyrosine kinase 4 (FLT4) ↑	Angiogenesis
	Growth differentiation factor 9 (GDF9 *) ↑	Required for ovarian folliculogenesis
	Nuclear receptor subfamily 1 group H member 3 (NR1H3) ↓	Regulates cholesterol metabolism and inflammation
	Wnt family member 3 (WNT3 *) ↑	Ligand in canonical Wnt signaling pathway
Immune system	Butyrophilin subfamily 3 member A1 (BTN3A1 *^&^) ↓	Regulates T-cell responses and the release of cytokines
	Butyrophilin subfamily 3 member A2 (BTN3A2 *^#&^) ↑	Regulates T-cell responses and the release of cytokines
	Butyrophilin subfamily 3 member A3 (BTN3A3 *^#&^) ↓	Regulates T-cell responses and the release of cytokines
	C1q and TNF related 4 (C1QTNF4 *^&^) ↑	Regulation of the inflammatory network
	CD276 molecule (CD276) ↓	Regulates T-cell responses and the release of cytokines
	CD40 molecule (CD40 *^&^) ↓	Regulation of inflammatory response
	Complement C4A (C4A) ↑	Component of the complement pathway. Mediator of local inflammatory processes
	Complement C4B (C4B *^#&^) ↓	Component of the complement pathway. Mediator of local inflammatory processes
	Interleukin 11 receptor subunit alpha (IL11RA) ↑	Receptor for interleukin 11. Controls the development of craniofacial bones and teeth
	Interleukin 21 receptor (IL21R) ↓	Receptor for interleukin-21
	Interleukin 33 (IL33 *) ↑	Regulates functions of various immune cells: T-helper type 2, macrophages, microglia, and NK cells
	Macrophage stimulating 1 receptor (MST1R *) ↑	Regulates cell survival, migration, and differentiation
Neurotransmission	5-hydroxytryptamine receptor 1D (HTR1D *) ↑	Neurotransmitter receptors
	Cholinergic receptor nicotinic alpha 3 subunit (CHRNA3) ↑	Neurotransmitter receptors
	Cholinergic receptor nicotinic beta 1 subunit (CHRNB1) ↓	Neurotransmitter receptors
	Dopamine receptor D2 (DRD2) ↓	Neurotransmitter receptors
	Neuropeptide Y receptor Y2 (NPY2R *) ↑	Receptor for neuropeptide Y and peptide YY
	Purinergic receptor P2X 2 (P2RX2) ↓	Receptor activated by extracellular ATP, mediates synaptic transmission between neurons
	Sodium voltage-gated channel alpha subunit 9 (SCN9A) ↑	Influx of Na^+^ ions provokes membrane depolarization
	Solute carrier family 1 member 6 (SLC1A6) ↓	Uptake of L-glutamate. Potentially plays a role in terminating the postsynaptic action of glutamate
	Solute carrier family 1 member 7 (SLC1A7 *) ↓	Uptake of L-glutamate. Potentially plays a role in terminating the postsynaptic action of glutamate
	Solute carrier family 12 member 5 (SLC12A5 *^#&^) ↑	Potassium-chloride cotransport in GABA-A and glycine neurons
Other receptors	G protein-coupled receptor 108 (GPR108 *) ↑	Regulation of immune response
	G protein-coupled receptor 27 (GPR27) ↑	Orphan receptor
	LDL receptor-related protein 4 (LRP4 *) ↓	Regulator of Wnt signaling, neuron differentiation
	LDL receptor-related protein 8 (LRP8) ↑	Participates in brain development
	Oxoglutarate receptor 1 (OXGR1 *) ↑	Inflammation regulation
	Vasoactive intestinal peptide receptor 2 (VIPR2) ↓	Receptor activated by the neuropeptides

**Table 4:** Summary of results of transcription analysis, including information on dataset, brain region, number of samples, and number of differentially expressed genes for male and female individuals.

GEO Dataset	Brain Region	Sample Size (Male) (MDD/Control)	Sample Size (Female) (MDD/Control)	|logFC| > 0.5 and *p*-Value < 0.05		|logFC| > 1 and Adjusted *p*-Value < 0.05	
				N of DEGs (Male)	N of DEGs (Female)	N of DEGs (Male)	N of DEGs (Female)
GSE80655	Anterior cingulate gyrus	18/21	6/3	70	416	0	0
	Dorsolateral prefrontal cortex	17/21	6/3	57	451	0	33
	Nucleus accumbens	16/20	6/2	193	469	1	5
GSE102556	Orbitofrontal cortex	13/13	12/9	44	147	0	9
	Dorsolateral prefrontal cortex	13/13	13/9	50	291	0	3
	Cingulate gyrus 25	3/8	9/7	593	378	51	24
	Anterior Insula	13/13	13/9	77	144	0	13
	Nucleus Accumbens	13/13	13/9	148	331	9	3
	Subiculum	12/12	12/7	439	390	54	28
GSE101521	Dorsolateral prefrontal cortex	19/23	11/6	389	58	6	0

## Data Availability

The original contributions presented in this study are included in the article/Supplementary Material. Further inquiries can be directed to the corresponding author.

## Supplementary Materials

The following supporting information can be downloaded at https://www.mdpi.com/article/10.3390/ijms26199557/s1.
